# Hybrid effects in field populations of the African monarch butterfly, *Danaus chrysippus* (L.) (Lepidoptera: Nymphalidae)

**DOI:** 10.1093/biolinnean/blab036

**Published:** 2021-04-26

**Authors:** David As Smith, Jon J Bennie, Ian J Gordon, Simon Martin, Piera Ireri, Kennedy S Omufwoko, Richard H Ffrench-Constant

**Affiliations:** 1 Natural History Museum, Eton College, Windsor SL4 6DW, UK; 2 Department of Geography, University of Exeter, Penryn Campus, Penryn TR10 9FE, UK; 3 Centre of Excellence in Biodiversity and Natural Resource Management, RN1, Huye Campus, Huye, Rwanda; 4 Institute of Evolutionary Biology, University of Edinburgh, Edinburgh EH1 3FL, UK; 5 Department of Zoological Sciences, Kenyatta University, Nairobi, P.O. Box 43844-00100, Kenya; 6 Mpala Research Centre (Princeton University), Nanyuki, P.O. Box 555-10400, Kenya; 7 Department of Ecology and Evolutionary Biology, Princeton University, Princeton, NJ 08544, USA; 8 Centre for Ecology and Conservation, University of Exeter, Penryn Campus, Penryn TR10 9FE, UK

**Keywords:** Asymmetric crossing, Bateson-Dobzhansky-Muller effect, body size, climate change, Haldane rule effect, heterosis, migration, non-random mating, reticulate evolution, speciation, wing length

## Abstract

Heterosis, Haldane and Bateson-Dobzhansky-Muller effects have been widely documented amongst a range of plants and animals. However, typically these effects are shown by taking parents of known genotype into the laboratory and measuring components of the F_1_ progeny under laboratory conditions. This leaves in doubt the real significance of such effects in the field. Here we use the well-known colour pattern genotypes of the African monarch or queen (*Danaus chrysippus*), which also control wing length, to test these effects both in the laboratory and in a contact zone in the field. By measuring the wing lengths in animals of known colour pattern genotype we show clear evidence for all three hybrid effects at the A and BC colour patterning loci, and importantly, that these same effects persist in the same presumptive F_1_s when measured in hybrid populations in the field. This demonstrates the power of a system in which genotypes can be directly inferred in the field and highlights that all three hybrid effects can be seen in the East African contact zone of this fascinating butterfly.

## INTRODUCTION

In crosses between genetically diverse parents, greater body size or fertility of an F_1_ progeny, such that it is either superior to the better of two parents (Mather & Jinks, 1982), or exceeds the mid-parent value (Falconer & Mackay, 1995), is known as heterosis. The phenomenon was first identified as hybrid vigour by both Darwin (1876) and Wallace (1889). Dobzhansky (1950, 1952) distinguished ‘euheterosis’ (in which Darwinian fitness, estimated as fecundity or longevity, is improved in F_1_ hybrids) from ‘luxuriance’, where the offspring are enhanced in a purely metric sense. ‘Euheterosis’ corresponds to ‘heterozygote advantage’ (Sheppard, 1967; Ford, 1971) or ‘overdominance’ (Wallace, 1970), whereas ‘luxuriance’ is synonymous with ‘hybrid vigour’ (Smith, 1980). As we have not estimated the fecundity or longevity of the F_1_ we are unable to distinguish ‘euheterosis’ from ‘luxuriance’; however, in crosses between wild, genetically deviant phenotypes, the latter is the likely model.

All genetic models for heterosis (Birchler *et al.*, 2006; Oakley *et al.*, 2015) hold that it is controlled by several loci, linked or unlinked, and attribute the heightened fitness of the F_1_, compared to its parents, to the masking of deleterious recessive alleles. According to one heterosis model—associative overdominance (Ohta & Kimura, 1970) —the masking of lethal or semi-lethal alleles is secured by recombination suppression and linkage disequilibrium. However, in practice, determining the precise genetic basis for heterosis is difficult because of undetected (or unexplained) epistasis and the many unknown genes that may contribute to the effect.

Haldane (1922) stated ‘*When in the F*_*1*_*offspring of two different animal races one sex is absent, rare or sterile, that sex is the heterozygous* [heterogametic] *one*’. Haldane found that in animals with an XY mechanism for sex determination (e.g. mammals and most insects), the sex adversely affected in the F_1_ was invariably the heterogametic (XY) male, whereas in ZW animals (e.g. birds and Lepidoptera) it was the female. Recognition of Haldane’s rule in dioecious plants such as *Silene* (Brothers & Delph, 2010), haplodiploid wasps (Koevoets & Beukeboom, 2009) and hermaphrodite pulmonates (Mollusca, Gastropoda) (Schilthuizen *et al.*, 2011) have hoisted its applicability towards universality (Coyne & Orr, 1989, 2004; Laurie, 1997); the Haldane rule ‘*represents a nearly obligatory first step in the evolution of postzygotic isolation*’ and, therefore, of speciation (Coyne & Orr, 1989). For a ZW butterfly, the cross *Danaus* [*chrysippus*] *klugii* (male) × *Danaus gilippus berenice* (female), which Ackery & Vane-Wright (1984) had suggested might be conspecific, is illustrative. The ZZ F_1_ males were viable whereas the ZW females, though equal in number, were inviable (Smith *et al.*, 2002); thus, the two are indeed distinct species.

Several genetic models have attempted to interpret the Haldane rule in diploid organisms (Charlesworth *et al.*, 1987; Hurst & Pomiankowski, 1991; Turelli & Orr, 2000; Tao & Hartle, 2003; Wang, 2003; Delph & Demuth, 2016); however, the dominance hypothesis (Haldane, 1932; Muller, 1942) is the most generally applicable. The dominance hypothesis postulates that heterogametic hybrids—the ZW female in all Lepidoptera (Sturtevant, 1915; Suomalainen *et al.*, 1973; Prowell, 1998; Traut *et al.*, 2008) —are affected by all Z-linked alleles, whether dominant or recessive, thus causing incompatibility when divergent alleles are brought together. Whereas ZZ (male) hybrids are only affected by dominant deleterious alleles, ZW (female) hybrids, which carry only one copy of any Z-linked gene, will be affected by all deleterious mutations regardless of dominance. As a result, hybrid inferiority is more evident in ZW females than ZZ males in butterflies (Turelli & Orr, 1995).

In large, outbred and more anciently-diverged populations, Bateson-Dobzhansky-Muller (BDM) effects (Bateson, 1909; Dobzhansky, 1933, 1936; Muller, 1939), otherwise known as ‘outbreeding depression’ or ‘underdominance’, is caused by genome-wide autosomal incompatibilities and equally affects both sexes (Wallace, 1889; Lynch, 1991; Orr & Turelli, 2001; Orr, 2005; Kirkpatrick & Barton, 2006). BDM is a model for an isolation mechanism which applies when both sexes of the F_1_ have lower fitness than the parents, whereas the Haldane rule is a trend which is observed only in the heterogametic sex.

The African monarch or queen butterfly, *Danaus chrysippus* (Linnaeus, 1758), comprises several named taxa which are largely geographically separated but cohabit and reticulate on a seasonal or semi-permanent basis through a large area of East-Central Africa. In previous papers, we have designated the area of hybridism as the contact zone (Smith *et al.*, 2016: fig. 1). At first description most of the geographical colour forms of *D. chrysippus* were described by their protologue authors (Linnaeus, 1758; Schreber, 1759; Cramer, 1777; Klug, 1845; Moore, 1883; Butler, 1886) as species. In more recent times, however, *D. chrysippus* populations in Africa have been split on the basis of characteristically predominant phenotypes (Smith *et al.*, 2019: fig. 2) which have been variously designated by the many authors involved as forms, races, polymorphs, genotypes, subspecies or semispecies of *D. chrysippus*. Genomic analyses have revealed that these polymorphisms are largely panmictic across much of the genome, with the exception of a few strongly differentiated ‘islands of divergence’. These include a broad region of suppressed recombination on chromosome 15 (chr15) containing the B and C colour patterning loci (hereafter termed the ‘BC locus’), and a similar region on chromosome 4 (chr4), putatively containing the A locus (Martin *et al.*, 2020). The precise geographical distributions of these divergent alleles, and frequency clines between them, remain to be described using molecular analyses across the range of the species.

**Figure 1.**
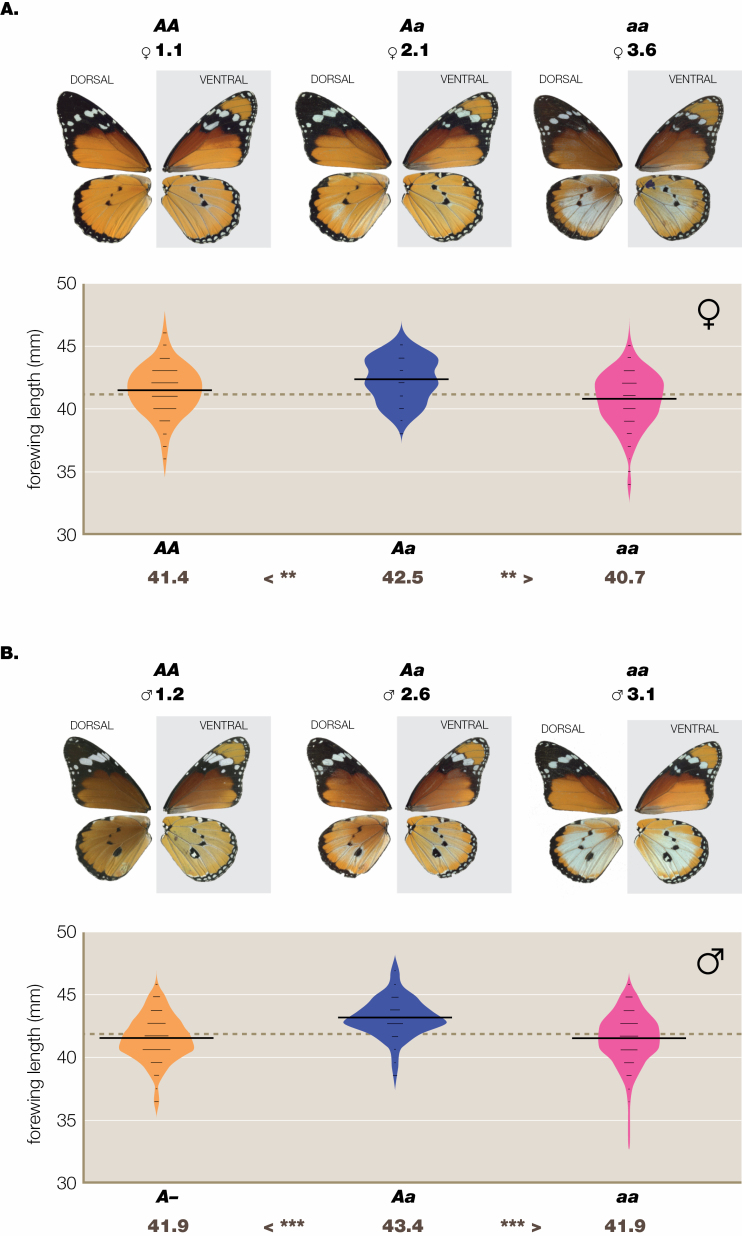
Heterosis at the A locus in laboratory crosses at Dar es Salaam, 1974–1975, data from DB2, (A) females and (B) males. The heterozygote *Aa* is significantly larger than both its homozygous parents, *AA* and *aa*, in both sexes. The variable ground colour of the wings (orange/brown) in this figure should be disregarded.

**Figure 2.**
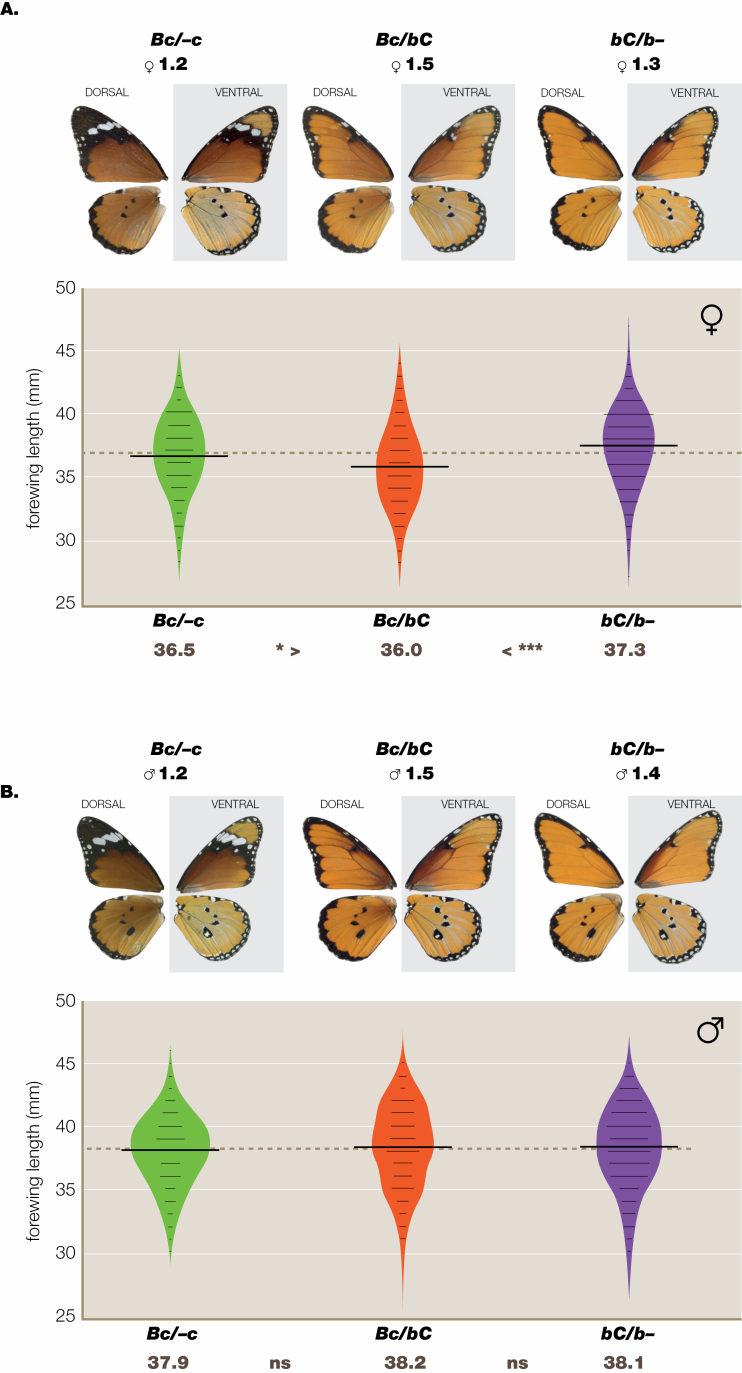
Haldane rule effect at the BC locus (Supporting Information, Table S8) in wild-collected butterflies from the contact zone (data from DB4). The presumptive cross is *orientis* (*Bc*/*Bc*) × *klugii* (*bC*/*bC*). In the F_1_, heterozygote (*Bc*/*bC*) females (A) are significantly smaller than both their homozygous parents, whereas among male F_1_ genotypes (B) there are no significant size differences.

It is important at this stage to point out to all readers that two changes to the nomenclature of *D. chrysippus* phenotypes are adopted in this paper (Table 1). First, the phenotype long known as dorippus (Klug, 1845), genotype *bC*/*bC* (Table 1), is re-named klugii (Butler, 1886), as recommended by Vane-Wright (2020). Second, as the name klugii is no longer available for the hybrid phenotype *Bc*/*bC*, the former klugii of Smith *et al.* (2019) is re-named ‘infumata’ (Aurivillius, 1899) (Table 1).

**Table 1.** The colour genotypes and phenotypes (numbers in bold) of *D. chrysippus* in Africa. The four morphs that are also considered as semi-species in the largely monomorphic parts of their range (outside the contact zone) are italicized whereas names of hybrid phenotypes are in Roman type enclosed within single quotes (Vane-Wright, 2020)

A locus	BC locus					
	*bc*/*bc* 1	*Bc*/*Bc* 2	*bC*/*bC* 3	*bC*/bc 4	*Bc*/*bC* 5	*Bc*/*bc* 6
*A*/*A***1**	1.1	1.2	1.3*	1.4^1^	1.5^2^	1.6^3^
	*chrysippus*	*orientis*	*klugii*	‘transiens’	‘infumata’	unnamed
*A*/*a***2**	2.1^4^§	2.2^4^§	2.3^5^	2.4^5^	2.5^5^	2.6^4^§
	‘semialcippus’	‘semialcippus’	‘semialbinus’	‘semialbinus’	‘semialbinus’	‘semialcippus’
*a*/*a***3**	3.1	3.2	3.3^6^	3.4^6^	3.5^6^	3.6
	*alcippus*	*alcippus*	‘albinus’	‘albinus’	‘albinus’	*alcippus*

Hybrids that occur in the contact zone alongside the four above-mentioned morphs are as follows: ^1^F_1_ hybrid form ‘transiens’ Suffert, 1900, from the cross 1.1 × 1.3; ^2^F_1_ hybrid f. ‘infumata’ Aurivillius, 1899, from the cross 1.2 × 1.3; ^3^unnamed F_1_ hybrid from cross 1.1 × 1.2; ^4^F_1_ hybrid f. ‘semialcippus’§, from crosses 1.1 × 3.1, 1.2 × 3.2 and 1.6 × 3.6; ^5^F_1_ hybrid f. ‘semialbinus’ Strand, 1910, from crosses 1.3 × 3.3, 1.4 × 3.4 and 1.5 × 3.5; ^6^F_2_ and backcross hybrid f. ‘albinus’ Lanz, 1896. The A locus on chr4 has two alleles, *A* (orange/brown hindwing) and *a* (white hindwing). The BC supergene on chr15 has three BC alleles: *bc*, as in *D.* [*c.*] *chrysippus*, **1.1** and *D.* [*c.*] *alcippus*, **3.1**–**3.2**; *Bc*, as in *D.* [*c.*] *orientis*, **1.2**; *bC*, as in *D.* [*c.*] *klugii*, **1.3.** A fourth possible allele, *BC*, is vanishingly rare. §Introduction of the name ‘semialcippus’ for the African hybrid (*A*/*a*), formerly named ‘alcippoides’, is new. The name ‘alcippoides’ was given by Moore (1883) to an Asian form; as this form may have either the *AA* or *Aa* genotype, it is inappropriate for the African *Aa* form.

The *D. chrysippus* polymorphism in East Africa is contentious because the butterfly is distasteful and therefore all morphs are aposematic. Although it does not exactly violate Ford’s commonly accepted definition of the term “*genetic polymorphism is the occurrence together in the same locality of two or more discontinuous forms of a species in such proportions that the rarest of them cannot be maintained by recurrent mutation*” (Ford, 1940), it disobeys the spirit. Ford’s definition, arguably, was envisaged to apply principally to polymorphic animals, such as snails and camouflaged moths, which display phaneropolymorphism (Huxley, 1955), individual morphs being either cryptic or Batesian mimics which are female-limited with free interbreeding among morphs. Although *D. chrysippus* polymorphisms have a geographic basis (Ford, 1940), they are seasonally variable and show a replicable pattern from year to year maintained by a migratory response to movements of the intertropical convergence zone (ITCZ) (Smith *et al.*, 1997). Partial assortative mating among morphs has been observed in regions of polymorphism (Gordon, 1984; Smith, 1984). In the light of what we now understand, *D. chrysippus* polymorphism is better described by the term ‘admixture polymorphism’, a term possibly first used by Wall (2000). As an alternative to Fordian polymorphism, admixture polymorphism is a gathering together on an ephemeral basis of distinct populations which are insufficiently diverged to be considered species and which may interbreed on a restricted and fortuitous basis. Notwithstanding the foregoing, Fordian polymorphism, whether transient or stable, has probably evolved from admixture polymorphism in *D. chrysippus*. As differential predation among morphs has been deduced from beak marks on the wings (Smith, 1979) and recapture data (Gordon *et al.*, 2010), polymorphic Müllerian mimicry, as in *Heliconius numata*(Jay *et al.*, 2021), has possibly evolved in *D. chrysippus* in some instances where polymorphism exists throughout the year.

Complete dominance has not evolved at any of the A and BC loci alleles which control aspects of colour pattern (Smith *et al.*, 2019; Martin *et al.*, 2020; Table 1; Fig. 1), suggesting that most hybridisms among the homozygous forms are relatively recent (< 2 Mya BP) events (Fisher, 1930; Clarke & Sheppard, 1960; Sheppard, 1967). Although all *D. chrysippus* morphs are inter-fertile (Poulton, 1925; Owen & Chanter, 1968; Clarke *et al.*, 1973; Smith, 1975; Gordon *et al.*, 1984), they also display either assortative mate choice (Gordon, 1984; Smith, 1984) or, when enforced by sex ratio differences, disassortative mating (Smith, 1973; Gordon *et al.*, 2014).

Forewing length in butterflies is a convenient proxy for body size as it is easily measured in both field and laboratory, and is constant throughout adult life—in contrast to body weight which fluctuates on a daily basis in response to various activities (Edgar & Culvenor, 1974; Edgar *et al.*, 1976; Boppré, 1983; Schulz *et al.*, 1993). Therefore, we here use wing length as a proxy for fitness to investigate the basis of different hybrid effects in crosses between morphs controlled by the A and BC colour pattern loci. Specifically, in the light of the lack of field-derived data showing heterosis and/or Haldane effects in natural populations, we use these well-defined colour pattern genotypes (Table 1; Supporting Information, Fig. S2) to test for these effects both in the laboratory and, more importantly, in the field itself. As we have not directly estimated the fitness of the F_1_ we are unable to disentangle ‘euheterosis’ from ‘luxurience’; however, in crosses between wild genetically distinct phenotypes, the latter is more probable.

## MATERIAL AND METHODS

The data for this study span 45 years from 1974 to 2019: the sampling area is detailed in Supporting Information (Table S1). Four databases (DB1-4) comprise 4685 butterflies (2088 males and 2597 females), including all 18 colour genotypes (Table 1; Supporting Information, Table S1). Because the B and C loci are linked within a supergene, within which recombination is suppressed, it is important to note that the number of genotypes is limited to 18, rather than the 27 expected from random assortment among three biallelic loci. DB1 comprises 1676 adults captured in the field at Dar es Salaam, measured, marked and then released. DB1 butterflies are not controlled for larval food plant but most will have fed in the wild upon *Calotropis gigantea*, with a minority, especially in the dry seasons, expected to use the xeromorphic climber *Tylophora stenoloba*, which is known to be an inferior host plant (Smith, 1978). DB2 consists of 792 butterflies from planned genetic crosses which were reared in the laboratory at Dar es Salaam and fed as larvae on *C. gigantea*. A collection (*N* = 1096) of wild-measured butterflies from various sites throughout the East African contact zone comprises DB3. Again, the larval food plant cannot be confirmed for this group but most will have fed in the wild on various species of *Gomphocarpus*. DB4 (*N* = 1121) comprises butterflies, either collected as eggs in the field, or reared from planned crosses in Kenya: all caterpillars were reared in the laboratory on *Asclepias curassavica*. As rearing conditions, laboratory (DB2 + DB4) vs. wild (DB1 + DB3), sex, larval food plant, collection site and season all influence adult body size, all four databases and the sexes within each database were analysed separately. Thus, experimental design exercises control for laboratory-reared vs. wild-collected, male vs. female and larval food plant, while statistical methods achieve a measure of control for seasonality, collection site and genotype.

The parameter used throughout the study is forewing length, measured to the nearest mm with a clear, plastic ruler. The dimension measured, marked as the line A-B in Supporting Information, Figure S1, is from the anterior thoracic attachment of the forewing (A) to its tip (B), which lies in space 7 (numerical system), midway between veins 7 and 8. Because 59.2% of the database comprises field-measured butterflies which, to comply with conservation considerations, were released after measuring and marking, measurement of forewing wing width, roundness and area (all desirable in future studies) were not attempted.

To control for seasonal differences in DB1, we analysed males and females separately using a general linear model (GLM) —here equivalent to a three-way ANOVA—with the A locus (coded as *A-*, *Aa* or *aa*), BC loci (coded as *Bc/bC*, *Bc/-c* or *bC/b-*) and month of collection (as a categorial variable) as independent variables. Models were produced with additive terms only, as well as with all combinations of interactions between factors. Separate models were also produced omitting month of collection as a factor. Model forms including full interaction terms, partial combinations of interactions and additive terms were compared using Akaike’s information criterion (AIC) values, and tested for significance against a null (intercept only) model using ANOVA. We used Q-Q plots to assess the normality of residuals, and post-hoc Tukey tests to define significant contrasts between sets to select the most parsimonious model in each case.

Other field-collected data sets (DB3 and DB4) were analysed separately for sex, and controlled for site as above, but not for season. Note that it is not possible to control effectively for season across sites in this data set as there is likely to be a different effect of season in different geographical regions and, in most data sets, data collection was only conducted during part of the year. Although it is apparent that the season of collection influences wing lengths in the Dar es Salaam data 1974–1975 (DB1) independently of genotypes, the frequency of genotypes also changed through the season. There are significant size differences between data sets/sites, independent of genotype, so site is always included as a covariate in the analysis. Comparing sites, there is no evidence that variance differs significantly among them. Notwithstanding the above caveats, the analysis benefits from large sample sizes and when all of the colour pattern genotypes are sampled sufficiently (see *Discussion*) the results are highly supported statistically. All analysis was carried out using R v.3.5.1 (R Core Team, 2018).

## RESULTS

Wing length in the wild-collected groups (DB1 + DB3) differ from the laboratory-reared groups (DB2 + DB4) by around 4 mm in both sexes (*P* = 4.42 × 10^–6^), presumably because laboratory-reared larvae are better nourished and protected. The wing length of males significantly exceeds that of females in both the laboratory-reared (*P* < 0.001) and wild-measured (*P* < 0.001) groups, as previously reported (Smith, 1980).

Seasonal variation in the wing lengths of males and females at Dar es Salaam from 1974–5 is shown in Supporting Information, Figure S2A. The beneficial effect of the main rains in April-May on size in the following months is clear. Although males are significantly larger than females in January-February and in July through to December, this size advantage at Dar es Salaam disappears from March-June. It is possible that the relative decline in male size in March-June is due to the arrival from the south of the *Bc*/-*c* (orientis) genotype which is significantly smaller than the *bC*/*b*- (klugii) genotype which it partially replaces (Supporting Information, Fig. S2C).

At the A locus (Supporting Information, Fig. S2B-C), frequency of the *Aa* genotype, though always low at Dar es Salaam, increases in June-August, especially in males (Supporting Information, Fig. S2C). At the BC supergene locus (Supporting Information, Fig. S2D-E), *bC*/*b*- (klugii) is the most abundant in January-March while *Bc*/-*c* (orientis) peaks in May-August. Whilst accepting that dispersal movements within a home range must be distinguished from genuine long-distance migration—in which movement to a new and distant home range is involved (Dingle, 2014) —these frequency changes (Supporting Information, Fig. S2) undoubtedly result from the latter (Smith & Owen, 1997; Smith *et al.*, 1997; Lushai *et al.*, 2003).

After controlling for seasonal changes in wing length, in laboratory-reared females at Dar es Salaam there are significant size differences between all genotypes at the A locus (Fig. 1; Supporting Information, Figs S3A, S5A; Table S2). The *A-* genotype (klugii and orientis) is larger than *aa* (alcippus) and the *Aa* genotype, being the largest, shows significant positive heterosis. In laboratory-reared males (Fig. 1; Supporting Information, Figs S3B, S5B; Table S2), the F_1_ genotype *Aa* is significantly larger than both parental genotypes; however, in this case, there is no significant difference between *A-* and *aa*. In wild-caught females at Dar es Salaam (Supporting Information, Figs S3C, S5C; Table S3), there are no significant differences among genotypes; however, as 96% of the sample was *A-*, scarcity of *Aa* and *aa* genotypes affects this result. In wild-caught males (Supporting Information, Figs S3D, S5D; Table S3), as *Aa* is significantly larger than *A-*, there is again evidence for positive heterosis in males. Supporting Information, Figure S3E, shows frequency distributions for forewing length in wild-caught females from data sets throughout the contact zone (DB4), with control for collecting site but not for season. Frequency distributions for forewing length in males (Supporting Information, Figs S3F, S5F; Table S4), with control for collecting sites, once more shows that the *Aa* class is larger than both parents but significantly so only compared to the *A-* parental class.

In summary, there is strong evidence for positive heterosis at the A locus in males, both at Dar es Salaam and in the wider contact zone. For females, evidence for heterosis that is statistically significant is confined to the laboratory-bred sample; however, the *Aa* and *aa* genotypes were inadequately represented in the field samples (Supporting Information, Tables S3-S4). Hence, we conclude that there is evidence for A locus heterosis in both sexes. The laboratory-reared broods suggest that the *A-* class is larger than the *aa* class. For both sexes, the A locus results are conservative since penetrance of the *a* allele in *Aa* heterozygotes is 62.5% (*N* = 56) in males and only 24.5% (*N* = 51) in females (Smith, 1998); thus, on average, only 43.5% of butterflies could be genotyped visually for the A locus. The interaction for penetrance at the A locus vs. sex is highly significant, χ ^2^_1_ = 14.78, *P* = 0.0001; however, there are no significant size differences or interactions with the BC loci (Smith, 1998: table 6).

In laboratory-reared females at Dar es Salaam (DB1, Fig. 2; Supporting Information, Figs S4A, S5G; Table S5) there are significant differences between genotypes *Bc*/-*c* (orientis) and both *Bc*/*bC* (‘infumata’) and *bC*/*b-*(klugii). Thus, klugii is the larger morph and the hybrid is intermediate. Among Dar es Salaam laboratory-reared males (DB1, Supporting Information, Figs S4B, S5H; Table S5) the heterozygote *Bc*/*bC* is significantly larger than its *Bc*/*-c* (orientis) parent but there is no clear hybrid effect. In wild-caught females from Dar es Salaam (DB2, Supporting Information, Figs S4C, S5I; Table S6), after controlling for seasonal changes in wing length, genotype *bC*/*b*- is significantly larger than its putative F_1_*Bc*/*bC*. The other putative parental class, *Bc*/-*c*, is intermediate and cannot be statistically distinguished from other genotypes. However, the size ranking *bC*/*b*- (klugii) > *Bc*/*bC* (‘infumata’) < *Bc*/-*c* (orientis) suggests relative unfitness in heterozygous females, which would be in agreement with the Haldane rule. This result is consistent with the breeding data in Supporting Information, Table S9, which show a significant shortage of *Bc*/*bC* females in the F_2_ in laboratory dihybrid crosses drawn from the same population that was field sampled. The shortfall of *Bc*/*bC* females (χ ^2^_1_ = 7.806, *P* = 0.002) and surplus of *Bc*/*bC* males (χ ^2^_1_ = 4.550, *P* = 0.033) are both significant and suggest heterozygote inviability in females and heterosis in males. Taken together both the wing length and viability data imply that hybrids which are heterozygous at the BC supergene conform to Haldane’s rule.

**Figure 3.**
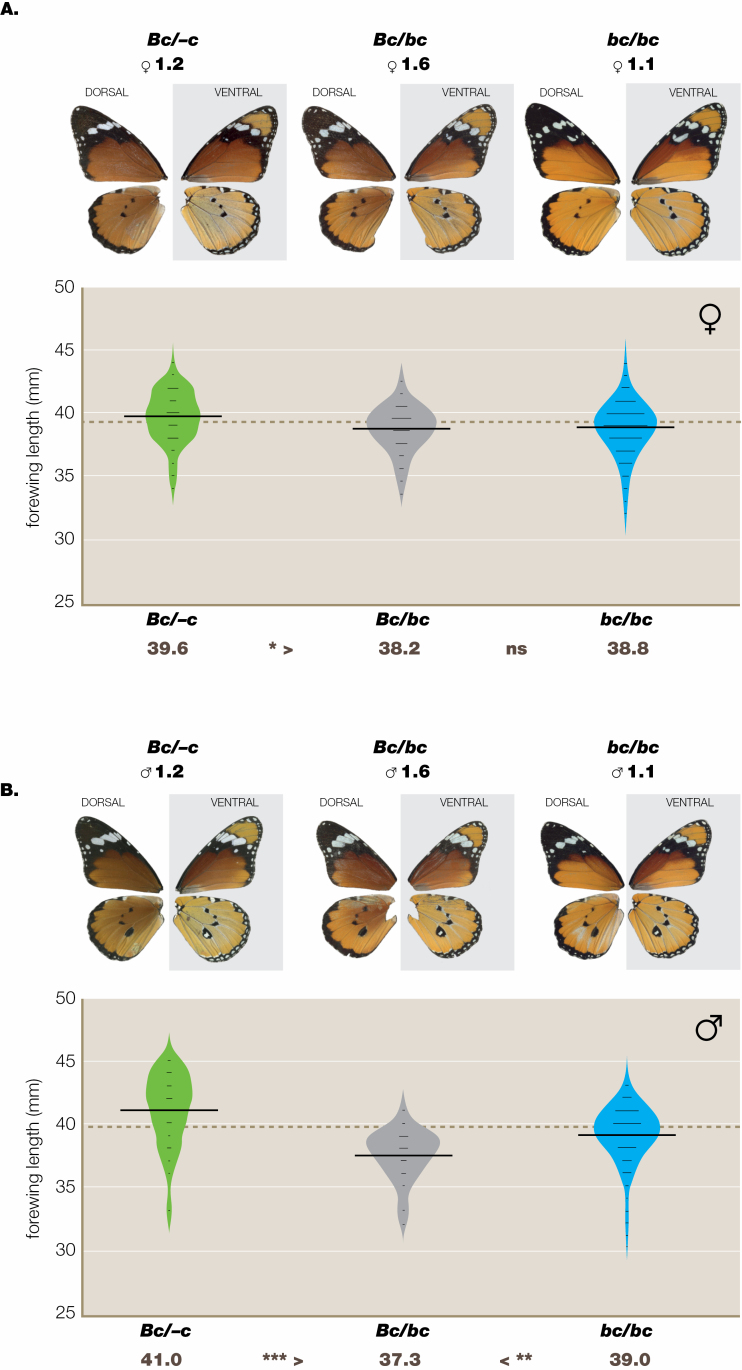
Bateson-Dobzhansky-Muller-like effect at the BC locus (Supporting Information, Table S7) in the presumptive cross *orientis* (*Bc*/*Bc*) × *alcippus*/*chrysippus* (*bc*/*bc*) in wild-collected eggs reared to adult in the laboratory (DB3). In both sexes (A, females; B, males) the F_1_ heterozygote is smaller than both its homozygous parents and significantly so in all cases except in the comparison female *bc*/*bc* and the F_1_*Bc*/*bc*.

Among females from wild-collected eggs throughout the hybrid zone (DB3, Supporting Information, Figs S4E, S5K, S, U; Table S7), in the putative cross klugii (*bC*/*b-*) × alcippus (*bc*/*bc*) the *bC*/*bc* (‘transiens’), offspring are significantly different from both parents and intermediate in size. In the putative cross *bC*/*b-* (klugii) × *Bc*/*-c* (orientis) there is a significantly lower wing length in F_1_ individuals identified as *Bc*/*bC* (‘infumata’) (Fig. 3A; Supporting Information, Figs S4E, S5M; Table S7) compared to both parents. As males (see below) are not similarly affected, this is again consistent with Haldane’s rule. In the putative cross orientis (*Bc*/-c) × alcippus (*bc*/*bc*) (Supporting Information, Fig. S5O) the F_1_*Bc*/*bc* females are smaller than both parents but significantly so only from the former.

In males (DB3, Fig. 3B; Supporting Information, Fig. S5R, T, V; Table S7) the *bC*/*bc* offspring are intermediate between the klugii and alcippus parents, and significantly smaller than the former. In the orientis × klugii cross there are no significant size differences between either parent and their F_1_ (Supporting Information, Fig. S5T). In the orientis × alcippus cross (Fig. 3B; Supporting Information, Figs S4F, S5P) the *Bc*/*bc* offspring are very significantly smaller than both putative parents. As both sexes are smaller than their presumptive parents, this is a BDM effect.

As heterozygotes at both B and C loci are only partially identifiable by sight in the field, the statistical conclusions are very conservative. Penetrance estimates obtained from breeding for *c* in *Cc* heterozygotes are: in *bC*/*bc* (‘transiens’), 0.514 (*N* = 1063); in *Bc*/*bC* (‘infumata’) 0.563 (*N* = 1209). Penetrance of *b* in *Bb*/*bc* heterozygotes cannot be reliably estimated from sight. As no interactions between A locus effects (Supporting Information, Fig. S5A-F) and BC effects (Supporting Information, Fig. S5G-V) were detected, the two effects are assumed to be additive.

There is ample evidence that the three morphs differ in wing length in the order klugii > orientis > alcippus in both sexes (Table 2). It seems that the BC supergene controls both wing morphology and colour pattern, as is known to be the case for supergenes in *Heliconius numata* (Cramer), *Melinaea* (Jones *et al.*, 2013) and *Papilio memnon* L. (Clarke *et al.*, 1968; Clarke & Sheppard, 1971). However, it is not clear from this study to what extent, if any, these differences in wing length might reflect in part either the migratory habit of the butterfly (Smith & Owen, 1997) or parasitism by *Spiroplasma* (Herren *et al.*, 2007). As both migration and parasitism are well known to affect body size in *D. plexippus* (Altizer *et al.*, 2000; Altizer & Davis, 2010; Altizer *et al.*, 2011; Dingle, 2014), it is our intention in future studies to investigate the possible influence of both factors in *D. chrysippus*.

**Table 2.** Size comparisons among the semi-species of *D. chrysippus*, females upper right, males lower left

	*bC*/*b-* (*klugii*)	*Bc*/*-c* (*orientis*)	*bc*/*bc* (*alcippus*)
*bC*/*b-* (*klugii*)	NC	*klugii* > orientis***	*klugii* > alcippus***
*Bc*/*-c* (*orientis*)	NS	NC	NS
*bc*/*bc* (*alcippus*)	*klugii* > alcippus***	*orientis* > alcippus***	NC

***, *P* < 0.001. NS, not significant. NC, no comparison.

The wild-caught samples from throughout the contact zone (DB4, Supporting Information, Figs S4E-F, S5K-P; Table S8) are controlled for collecting site but not for season. The frequency distributions for forewing length between BC loci in wild-caught males from all data sets (Supporting Information, Figs S4H, S5R, T, V) show no significant differences. For females, the putative cross *klugii* (*bC*/*b-*) × *orientis* (*Bc*/*-c*) gives ‘infumata’ (*Bc*/*bC*) offspring (Supporting Information, Figs S4G, S5S; Table S8) which are significantly smaller than both parents; however, male hybrid offspring from the same cross (Supporting Information, Figs S4H, S5T; Table S8) are not similarly affected. This is consistent with Haldane’s rule.

## DISCUSSION

Here we have used the well described colour pattern loci of the African monarch to test for different hybrid effects both in controlled laboratory crosses and in the presumptive F_1_ animals captured in contact zones in the field. Strikingly, we find evidence for all three anticipated effects: heterosis (A locus), BDM incompatibilities (BC locus) and sex-specific effects consistent with Haldane’s rule (BC locus) can clearly be seen in different crosses both in the laboratory and more importantly in the field. This leads to several important conclusions. First, by using clearly defined autosomal markers for the separate semi-species we can document hybrid effects in the field that have previously only been seen in the laboratory (Smith, 1980), raising the possibility that similar approaches could be used in other animals and plants. Second, our data suggest that the colour pattern loci affect both colour and body size and that it is these autosomal loci themselves that are largely responsible for the hybrid effects seen.

Whatever the outcome of the ongoing discussion regarding the subspecific divisions of *D. chrysippus* and their nomenclature (Vane-Wright & John, 2019; Vane-Wright, 2020), the body size differences of hybrid offspring described here, compared to their homozygous parents, are at once atypical for a polymorphism but conform to expectation if disruptive selection is (or has been) underway. Such differences among homozygous parents and their heterozygous progeny are commonplace when hybrids between ‘ecotypes’ that have been selected ecologically in different environments, such as in three-spine sticklebacks, *Gasterosteus aculeatus* L. (Gow *et al.*, 2007), are adversely selected whenever their parents interbreed. Owen & Chanter (1968) noted that in *D. chrysippus* in Uganda, *Aa* hybrids at the A colour gene locus were under-represented in wild populations compared to their presumptive *AA* and *aa* parents, again suggesting disruptive selection against heterozygotes.

These hybrid effect findings have implications for the hypothesis of ongoing speciation among the several forms of *D chrysippus* in Africa, which occupy substantially different ranges, where each colour form is close to monomorphic. It seems likely that the colour pattern variation in *D. chrysippus* was evolved in allopatry and in response to differing environments (Smith *et al.*, 2016, 2019). As the species is notoriously migratory (Smith & Owen, 1997), especially the males, both sex ratio and morph composition at any single site are in constant flux (Supporting Information, Fig. S2B-E), mostly in response to the north-south oscillations of the ITCZ. Therefore, both polymorphism and sex-ratio variation in a given place are to some extent artefacts created by the high mobility of the species and more rapid dispersal of males. Because all the geographical forms retain the ability to mate and bear fertile offspring in the contact zone, numerous hybrid colour forms occur.

Whereas, on the isolation by distance principle (Wright, 1943), marker colour genes have reached all quarters of the African continent and even far beyond, e.g. Sri Lanka (Vane-Wright, 2020), the part of East Africa we call the contact zone (Smith *et al.* 2019; Fig. S6) is an area of permanent admixture polymorphism where the frequency of each semi-species varies seasonally (Fig. 2B), in the main caused by migration (Smith & Owen, 1997). This is, emphatically, not to contend that no stragglers will remain outside their usual geographical limits and possibly interbreed with long-term residents within the contact zone. It is within this contact zone that, when hybridizing with neighbouring aliens, sporadically separated semi-species have each left their marks in the form of heterosis, Haldane’s rule and BDM effects. Heterosis occurs in the cross *A-* (*klugii* + *orientis*) × *aa* (*alcippus*) (Fig. 1); Haldane effects are associated with the crosses klugii × orientis and orientis × alcippus (Fig. 2); a full BDM effect is found only in the presumptive cross *Bc*/*-c* (orientis) × *bc*/*bc* (chrysippus + alcippus) (Fig. 3); the latter most, especially, suggests either that incipient speciation is ongoing, or alternatively, that it might have occurred in the recent past and been subsequently interrupted or reversed (Smith, 1980).

An overall interpretation of the various hybrid effects suggests, firstly, that a relatively ancient split occurred between (*klugii* + *orientis*) and (*alcippus* + *chrysippus*), a divergence which manifests as heterosis and BDM effects in crosses between them. Secondly, a later divergence in East Africa which involved, in particular, *klugii* and *orientis*, is indicated by Haldane effects. Our findings also have implications for understanding the mechanisms underlying Haldane’s rule. There is convincing evidence that Haldane’s rule can usually be explained by the exposure of recessive Z-linked (or X-linked) incompatibilities in the heterogametic sex (Coyne, 2018) – originally coined the ‘large X effect’ (Coyne & Orr, 2004). However, we have here defined ‘hybrids’ purely by being heterozygous at autosomal markers, with no evidence for the population origin of the Z and W chromosomes in the crosses assessed. Moreover, the Z chromosome shows minimal genetic differentiation among semi-species (Martin *et al.*, 2020), implying that it is largely homogenized by gene flow, and is therefore unlikely to harbour BDM incompatibility loci (Bank *et al.*, 2012). Therefore, the reduced hybrid female fitness associated with heterozygosity at the BC supergene could either be explained through an interaction with the W chromosome (which has yet to be sequenced) or must be unrelated to the fact that females are heterogametic. Further work will be needed to resolve this.

Previous studies of all three effects on field populations of animals and plants have relied upon isolating strains from the field and then crossing them in the laboratory. Here we have done the same but have extended our work to test for the actual significance of these interactions in the field itself. Thus, by comparing laboratory crosses of known genotypes with data from presumptive F_1_s in the field we have been able to confirm that these effects are still (highly) significant under field conditions.

## SUPPORTING INFORMATION

Additional Supporting Information may be found in the online version of this article at the publisher’s web-site:

**Table S1.** Wing length xlsx files available on request. Table of all databases used in this study and their associated codes.

**Table S2.** A, probabilities (*p*) of difference among wing lengths (mm) of genotypes at the A locus of *D. chrysippus* laboratory-reared on *C. gigantea* at Dar es Salaam (1974-1975). DB2, Supporting Information, Figure 5A-B. B, raw data for Supporting Information, Table S2A.

**Table S3.** A, probabilities (*p*) of difference among wing lengths (mm) of genotypes at the A locus of *D. chrysippus* wild caught at Dar es Salaam (1974-1975), DB1, Supporting Information, Figure 5C-D. B, raw data for Supporting Information, Table S3A.

**Table S4.** A, probabilities (*p*) of difference among wing lengths (mm) of genotypes at the A locus of *D. chrysippus* wild caught throughout the contact zone, DB4, Supporting Information, Figure 5E-F. B, raw data for Supporting Information, Table S4A.

**Table S5.** A, probabilities (*p*) of difference among wing lengths (mm) of genotypes at the BC locus of *D. chrysippus* laboratory-reared on *C. gigantea* at Dar es Salaam (1974-1975), DB2, Supporting Information, Figure 5G-H. B, raw data for Supporting Information, Table S5A.

**Table S6.** A, probabilities (*p*) of difference among wing lengths (mm) of genotypes at the BC locus of *D. chrysippus* caught wild at Dar es Salaam (1974-1975), DB1 in part and DB4, Supporting Information, Figure 5I-J. B, raw data for Supporting Information, Table S6A.

**Table S7.** A, probabilities (*p*) of difference among wing lengths (mm) of genotypes at the BC locus of *D. chrysippus* wild-collected eggs, laboratory-reared reared on *Asclepias curassavica* at Nairobi (1985-1986), DB3, Supporting Information, Figure S5K-P. Presumptive crosses are *klugii* (*bC*/*b-*) × *alcippus* (*bc*/*bc*) (S5K-L), *klugii* × *orientis* (*Bc*/*-c*) (S5M-N), *orientis* × *alcippus* (S5O-P). B, raw data for Supporting Information, Table S7A.

**Table S8.** A, probabilities (*p*) of difference among wing lengths (mm) of genotypes at the BC locus of *D. chrysippus* wild caught throughout the contact zone, DB4, Supporting Information, Figures S5Q-V. Presumptive crosses are *klugii* (*bC*/*b-*) × *alcippus* (*bc*/*bc*) (Q-R), *klugii* × *orientis* (*Bc*/*-c*) (S5S-T), *orientis* × *alcippus* (S5U-V). B, raw data for Supporting Information, Table S8A.

**Table S9.** F_2_ offspring (expected numbers in parenthesis) from 13 *Bc*/*bC* × *Bc*/*bC* crosses at Dar es Salaam, Tanzania, 1975. Data from Smith (2014).

**Figure S1.** Forewing length in *D. chrysippus*. The blue line A-B is the parameter measured. The numbering system for veins and spaces follows Higgins & Riley (1970).

**Figure S2.** A, seasonal variation in wing lengths of *D. chrysippus* – males (blue) and females (brown). Error bars show two standard errors of the mean. B, seasonal frequencies of A locus genotypes in females. C, seasonal frequencies of A locus genotypes in males. D, seasonal frequencies of BC genotypes in females. E, seasonal frequencies of BC genotypes in males. All the data relate to butterflies wild caught, marked and released on the university campus at Dar es Salaam in 1974-1975. Genotype and phenotype (Table 1) of the butterflies figured are as follows: *A-*, 1.1 ♀; *Aa*, 2.1 ♀; *aa*, 3.1. ♂; *Bc*/*bc*, 1.6 ♂; *Bc*/*bC*, 1.5, ♀; *bC*/*bc*, 1.4, ♂; *bC*/*b-*, 1.3, ♀; *Bc*/*-c*, 1.2, ♂; *bc*/*bc*, 1.1, ♀.

**Figure S3.** Beanplots showing frequency distributions of forewing lengths (mm) associated with genotypes at the A locus at Dar es Salaam. A, laboratory-reared females (DB2). B, laboratory-reared males (DB2). C, wild-caught females (DB1). D, wild-caught males (DB1). E, wild-caught females, (F) wild-caught males from DB4. C and D controlled for seasonal change, E and F controlled for collecting sites but not for seasonal change.

**Figure S4.** Beanplots showing frequency distributions of forewing lengths (mm) associated with genotypes at the BC locus. A. Lab-reared females from Dar es Salaam (DB2), controlled for food-plant. B. Lab-reared males from Dar es Salaam (DB2), controlled for food-plant. C. Wild-caught females from Dar es Salaam (DB1). D. Wild-caught males from Dar es Salaam (DB1). E. Wild-caught females from throughout the contact zone (DB4). F. Wild-caught males from throughout the contact zone (DB4). G. Female samples reared from wild-collected eggs (DB3), controlled for food-plant. H. Male samples reared from wild-collected eggs (DB3), controlled for food-plant.

**Figure S5.** Graphs showing the observed wing lengths of *D. chrysippus* parents and, in laboratory crosses, F_1_ hybrids. The sexes are shown separately throughout. In wild populations the F_1_ crosses are presumptive rather than actual. Points in blue show, on the left, the mean values of the two parent genotypes and, on the right, the F_1_. Mid-parent values are marked as horizontal lines in blue. Statistical significance of size differences: * *P* < 0.05, ** *P* < 0.01, *** *P* < 0.001, **** *P* < 0.0001. Differences not marked as statically significant might in some cases be so if sample sizes were larger. A, female and (B) male genotypes, *A*-, *aa* (parents) and *Aa* (F_1_) in laboratory-reared crosses at Dar es Salaam, DB2, controlled for food plant. C, female and (D) male genotypes, *A*-, *aa* (parental genotypes) and *Aa* (F_1_ genotype) in a wild population at Dar es Salaam, DB1. E, female and (F) male genotypes, *A*- *aa* (parental genotypes) and *Aa* (F_1_ genotype) in wild populations through the contact zone, DB3, controlled for collecting site. G, female and (H) male genotypes, *Bc*/*-c*, *bC*/*b-* (parents) and *Bc*/*bC* (F_1_) in laboratory-reared crosses at Dar es Salaam, DB2, controlled for food plant. I, female and (J) male genotypes, *Bc*/*-c*, *bC*/*b-* (parental genotypes) and *Bc*/*bC* (F_1_ genotype) in a wild population at Dar es Salaam, DB1. K, female and (L) male genotypes, *bC*/*b-*, *bc*/*bc* (parental genotypes) and *bC*/*bc* (F_1_ genotype) in wild populations through the contact zone, DB4. M, female and (N) male genotypes, *Bc*/*-c*, *bC*/*b-* (parental genotypes) and *Bc*/*bC* (F_1_ genotype) in wild populations through the contact zone, DB4. O, female and (P) male genotypes, *Bc*/*-c*, *bc*/*bc* (parental genotypes) and *Bc*/*bc* (F_1_ genotype) in wild populations through the contact zone, DB4. Q, female and (R) male genotypes, *bC*/*b-*, *bc*/*bc* (parental genotypes) and *bC*/*bc* (F_1_ genotype) in laboratory-reared butterflies reared from wild collected eggs in the contact zone, DB3 controlled for food plant. S, female and (T) male genotypes, *Bc*/*-c*, *bC*/*b-* (parental genotypes) and *Bc*/*bC* (F_1_ genotype) in laboratory-reared butterflies reared from wild collected eggs in the contact zone, DB3, controlled for food plant. U, female and (V) male genotypes, *Bc*/*-c*, *bc*/*bc* (parental genotypes) and *Bc*/*bc* (F_1_ genotypes) in laboratory-reared butterflies reared from wild collected eggs in the contact zone, DB3, controlled for food plant.

**Figure S6.** A, African vegetation at the maximum extent of Pleistocene glaciation, 18 Kya BP. Q, Mount Camaroun; (R) Ethiopian Highlands; (S) Kenya Highlands; (T) Tanzanian Highlands. B, African vegetation from ~15-5 Kya BP in the Holocene, known as the African Humid Period (AHP). A, River Niger; (B) River Benue; (C) River Nile; (D) River Congo; (E) River Zambezi; (F) Araoune Basin; (G) Ténére Basin; (H) Lake MegaChad; (I) Lake Tana; (J) Sudan Swamp; (K) Lake Turkana; (L) Lake Victoria; (M) Lake Tanganyika; (N) Lake Malawi; (O) Okavango Delta; (P) Lake Zaire. Vegetation maps for former glacial periods would resemble Figure 6A. A vegetation map for the last interglacial (Eemian) Period, 130-115 Kya BP (marine isotope stage 5e), is virtually identical to Figure 6B, as are vegetation reconstructions for many former interglacial and interstadial periods in the Pleistocene.

blab036_suppl_Supplementary_Material

## FUNDING

Funded by a grant BB/H014268/1 to R.ff-C from the Biotechnology and Biological Sciences Research Council or BBSRC
