# Supplementary material for: Hybrid effects in field populations of the African monarch butterfly, Danaus chrysippus (L.) (Lepidoptera: Nymphalidae)
Source: Biol J Linn Soc Lond. 2021 Apr 26;133(3):671–84. doi: 10.1093/biolinnean/blab036 (PMC8444992; doi:10.1093/biolinnean/blab036)
Supplement: blab036_suppl_Supplementary_Material [file blab036_suppl_Supplementary_Material.docx]

**Supplementary Information**

**Supplementary Tables**

_________________________________________________________________________________________

Table S1. Wing length xlsx files available on request

__________________________________________________________________________________________

Database (DB)1, wild-measured (WM) adults, Dar es |Salaam, Tanzania, 1974-5, *n* = 1676

AQ1 females

AQ2 males

DB2, laboratory-reared (LR) families, Dar es Salaam, Tanzania, 1974-5, *n* = 792

AQ3 females from mixed-sex families

AQ4 males from mixed-sex families

AQ5 females from male-killer (MK) families

DB3, *n* = 1121, from wild-collected (WC) eggs and LR crosses, reared on *Asclepias curassavica*

AQ7 both sexes, Kasarani nr. Nairobi, Kenya, 2005, 2009-10.

AQ8 females, Athi River, Nairobi, Kenya, 1985-9.

AQ9 males, Athi River, Nairobi, Kenya, 1985-9.

AQ11 bisexual families, W-linked for BC colour genes, both sexes, Nairobi, Kenya, 1985-9.

AQ12 bisexual families, segregation independent for sex and colour, both sexes, Nairobi, Kenya, 1985-9.

AQ13 eggs/larvae, both sexes, Cape Coast, Ghana, 1985.

AQ14 eggs/larvae, both sexes, Harare, Zimbabwe, 1989.

DB4, *n* = 1096, WM adults from various sites throughout the contact zone

AQ6 both sexes, Kitengela nr. Nairobi, Kenya, 2014-5.

AQ16 both sexes, from Chiromo, Tana River, Amboseli, Bamburi, Galana River, Gede, Watamu and

Lolldaiga, all Kenya

AQ17 both sexes, miscellaneous sites (Dar es Salaam, Tanzania; Mangochi, Malawi; Makerere, Uganda;

Cape Coast, Ghana; Nyamata, Rwanda), various dates.

AQ19 WM, both sexes, Nyamata, Rwanda, 2016-7.

AQ20 WM, all female, Nguruman, Kenya, 2005.

__________________________________________________________________________________________

Table S2. A. Probabilities (*p*) of difference among wing lengths (mm) of genotypes at the A locus of *D. chrysippus* lab-reared on *Calotropis gigantea* at Dar es Salaam (1974-5). DB2, SI Fig. 5.A-B

___________________________________

1. *Aa* *aa*

___________________________________

*A-* NC ** ***

*Aa* *** NC **

*aa* ns *** NC

___________________________________

Female comparisons top right, males bottom left. * 0.05 > *p* > 0.01; ** 0.01 > *p* > 0.001; *** *p* < 0.001; **** *p* < 0.0001; ns, not significant; NC, no comparison.

_________________________________________________

Table S2. B. Raw data for Table S2. A

_________________________________________________

Genotypes *A-* *Aa* *aa*

**Females**

Mean wing length (mm) 41.4 42.5 40.7

Standard error (mm) 0.119 0.273 0.181

*N* 258 37 142

**Males**

Mean wing length (mm) 41.9 43.4 41.9

Standard error (mm) 0.135 0.188 0.170

*N* 159 61 135

_________________________________________________

Table S3. A. Probabilities (*p*) of difference among wing lengths (mm) of genotypes at the A locus of *D. chrysippus* wild caught at Dar es Salaam (1974-5), DB1, SI Fig. 5.C-D

___________________________________

*A-* *Aa* *aa*

___________________________________

*A-* NC ns ns

*Aa* * NC ns

*aa* ns ns NC

___________________________________

Female comparisons top right, males bottom left. * 0.05 > *p* > 0.01; ** 0.01 > *p* > 0.001; *** *p* < 0.001; **** *p* < 0.0001; ns, not significant; NC, no comparison.

_________________________________________________

Table S3. B. Raw data for Table S3. A

_________________________________________________

Genotypes *A-* *Aa* *aa*

**Females**

Mean wing length (mm) 36.0 36.2 37.7

Standard error (mm) 0.124 0.690 1.176

*N* 650 17 8

**Males**

Mean wing length (mm) 37.9 38.8 37.0

Standard error (mm) 0.107 0.258 1.20

*N* 891 113 10

_________________________________________________

Table S4. A. Probabilities (*p*) of difference among wing lengths (mm) of genotypes at the A locus of *D. chrysippus* wild caught throughout the contact zone, DB4, SI Fig. 5.E-F

___________________________________

*A-* *Aa* *aa*

___________________________________

*A-* NC ns ns

*Aa* * NC ns

*aa* ns ns NC

___________________________________

Female comparisons top right, males bottom left. * 0.05 > *p* > 0.01; ** 0.01 > *p* > 0.001; *** *p* < 0.001; **** *p* < 0.0001; ns, not significant; NC, no comparison.

_________________________________________________

Table S4. B. Raw data for Table S4. A

_________________________________________________

Genotypes *A-* *Aa* *aa*

**Females**

Mean wing length (mm) 37.0 37.9 38.1

Standard error (mm) 0.089 0.508 0.262

*N* 1306 59 68

**Males**

Mean wing length (mm) 38.2 39.3 38.2

Standard error (mm) 0.093 0.242 0.300

*N* 1127 134 78

_________________________________________________

Table S5 A. Probabilities (*p*) of difference among wing lengths (mm) of genotypes at the BC locus of *D. chrysippus* lab-reared on *Calotropis gigantea* at Dar es Salaam (1974-5), DB2, SI Fig. 5.G-H

___________________________________

*Bc*/*-c* *Bc*/*bC* *bC*/*b-*

___________________________________

*Bc*/*-c* NC ** ***

*Bc*/*bC* * NC ns

*bC*/*b-* ns ns NC

___________________________________

Female comparisons top right, males bottom left. * 0.05 > *p* > 0.01; ** 0.01 > *p* > 0.001; *** *p* < 0.001; **** *p* < 0.0001; ns, not significant; NC, no comparison.

_________________________________________________

Table S5. B. Raw data for Table S5. A

_________________________________________________

Genotypes *Bc*/*-c* *Bc*/*bC* *bC*/*b-*

**Females**

Mean wing length (mm) 41.0 41.3 41.7

Standard error (mm) 0.130 0.201 0.177

*N* 172 140 125

**Males**

Mean wing length (mm) 41.8 42.3 42.2

Standard error (mm) 0.176 0.134 0.242

*N* 100 175 80

_________________________________________________

Table S6. A. Probabilities (*p*) of difference among wing lengths (mm) of genotypes at the BC locus of *D. chrysippus* caught wild at Dar es Salaam (1974-5), DB1 in part, SI Fig. 5.I-J

___________________________________

*Bc*/*-c* *Bc*/*bC* *bC*/*b-*

___________________________________

*Bc*/-c NC ns ns

*Bc*/*bC* ns NC *

*bC*/*b-* ns ns NC

___________________________________

Female comparisons top right, males bottom left. * 0.05 > *p* > 0.01; ** 0.01 > *p* > 0.001; *** *p* < 0.001; **** *p* < 0.0001; ns, not significant; NC, no comparison.

_________________________________________________

Table S6. B. Raw data for Table S6. A

_________________________________________________

Genotypes *Bc*/*-c* *Bc*/*bC* *bC*/*b-*

**Females**

Mean wing length (mm) 36.1 35.6 36.4

Standard error (mm) 0.854 0.402 0.194

*N* 4 56 251

**Males**

Mean wing length (mm) 37.8 38.0 38.2

Standard error (mm) 1.167 0.286 0.160

*N* 9 104 384

_________________________________________________

Table S7. A. Probabilities (*p*) of difference among wing lengths (mm) of genotypes at the BC locus of *D. chrysippus* wild-collected eggs, lab-reared reared on *Asclepias curassavica* at Nairobi (1985-6), DB3, SI Fig. 5.K-P. Presumptive crosses are *klugii* (*bC*/*b-*) × *alcippus* (*bc*/*bc*) (K-L), *klugii* × *orientis* (*Bc*/*-c*) (M-N), *orientis* × *alcippus* (O-P)

_________________________________________________________

*bc*/*bc* *bC*/*bc* *bC*/*b-* *Bc*/*bC* *Bc*/*-c* *Bc*/*bc*

_________________________________________________________

*bc*/*bc* NC * **** ns ns ns

*bC*/*bc* ns NC ** *** ns ***

*bC*/*b-* **** *** NC ns ns ****

*Bc*/*bC* ns ns ns NC ** ns

*Bc*/*-c* *** ns ns ns NC *

*Bc*/*bc* ** **** **** ns **** NC

_________________________________________________________

Female comparisons top right, males bottom left. * 0.05 > *p* > 0.01; ** 0.01 > *p* > 0.001; *** *p* < 0.001; **** *p* < 0.0001; ns, not significant; NC, no comparison.

_______________________________________________________________________

Table S7. B. Raw data for Table S7. A

_______________________________________________________________________

Genotypes *bc*/*bc* *bC*/*bc* *bC*/*b-* *Bc*/*bC* *Bc*/*-c* *Bc*/*bc*

**Females**

Mean wing length (mm) 38.8 39.6 40.6 37.8 39.6 38.2

Standard error (mm) 0.15 0.20 0.22 0.31 0.28 0.21

*N* 193 221 130 55 54 74

**Males**

Mean wing length (mm) 39.0 39.7 41.3 40.0 41.0 37.3

Standard error (mm) 0.19 0.20 0.26 1.00 0.57 0.36

*N* 139 129 64 2 27 33

______________________________________________________________________

Table S8. A. Probabilities (*p*) of difference among wing lengths (mm) of genotypes at the BC locus of *D. chrysippus* wild caught throughout the contact zone, DB4, SI Fig. 5.Q-V. Presumptive crosses are *klugii* (*bC*/*b-*) × *alcippus* (*bc*/*bc*) (Q-R), *klugii* × *orientis* (*Bc*/*-c*) (S-T), *orientis* × *alcippus* (U-V).

_________________________________________________________

*bc*/*bc* *bC*/*bc* *bC*/*b-* *Bc*/*bC* *Bc*/*-c* *Bc*/*bc*

_________________________________________________________

*bc*/*bc* NC ns ns * ns ns

*bC*/*bc* ns NC ns *** ns ns

*bC*/*b-* ns ns NC *** * ns

*Bc*/*bC* ns ns ns NC * ns

*Bc*/*-c* ns ns ns ns NC ns

*Bc*/*bc* ns ns ns ns ns NC

_________________________________________________________

Female comparisons top right, males bottom left. * 0.05 > *p* > 0.01; ** 0.01 > *p* > 0.001; *** *p* < 0.001; **** *p* < 0.0001; ns, not significant; NC, no comparison.

_______________________________________________________________________

Table S8. B. Raw data for Table S8. A

_______________________________________________________________________

Genotypes *bc*/*bc* *bC*/*bc* *bC*/*b-* *Bc*/*bC* *Bc*/*-c* *Bc*/*bc*

**Females**

Mean wing length (mm) 36.7 37.1 37.3 36.0 36.5 36.7

Standard error (mm) 0.269 0.181 0.135 0.223 0.207 0.532

*N* 154 274 508 238 237 22

**Males**

Mean wing length (mm) 37.8 38.3 38.1 38.2 37.9 38.5

Standard error (mm) 0.284 0.176 0.138 0.120 0.181 0.645

*N* 80 254 504 137 336 28

______________________________________________________________________

_________________________________________________________________

Table S9. F_2_ offspring (expected numbers in parenthesis) from 13 *Bc*/*bC* × *Bc*/*bC*

crosses at Dar es Salaam, Tanzania, 1975. Data from Smith, 2014.

________________________________________________________________

Sex *bC*/*bC* *Bc*/*bC* *Bc*/*Bc* *N*

________________________________________________________________

Male 29 (34.625) 87 (69.250) 49 (34.625) 165

Female 34 (34.625) 46 (69.250) 32 (34.625) 112

*N* 63 133 81 277

________________________________________________________________

Item tested χ^2^ d.f. *p*

________________________________________________________________

Segregation at the BC loci, *H*_0_ 1:2:1 2.776 2 0.250

Segregation for sex, *H*_0_ 1:1 10.141 1 < 0.001

Sex-B locus interaction 6.412 1 0.011

Sex-C locus interaction 0.271 1 0.603

Total 19.601 5 < 0.001

________________________________________________________________

Expected numbers are generated from independent expectations (*H*_0_) of a 1:1

segregation for sex and a 1:2:1 segregation for genotype (assuming complete

linkage between the B and C loci).

________________________________________________________________

**Supplementary figures**


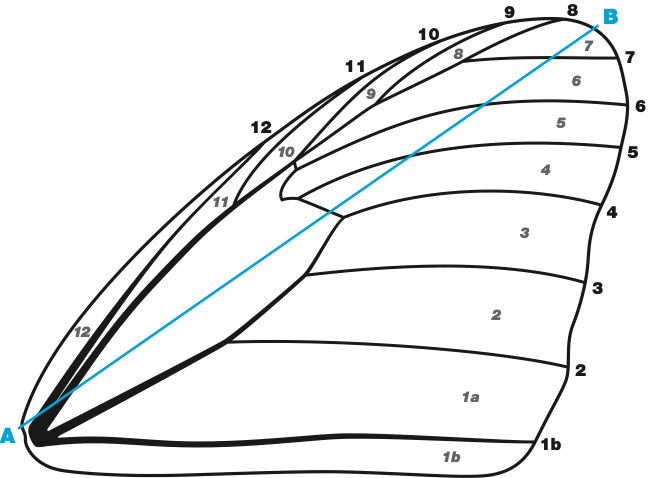


Figure S1. Forewing length in *Danaus chrysippus*. The blue line A-B is the parameter measured. The numbering system for veins and spaces follows Higgins and Riley (1970).


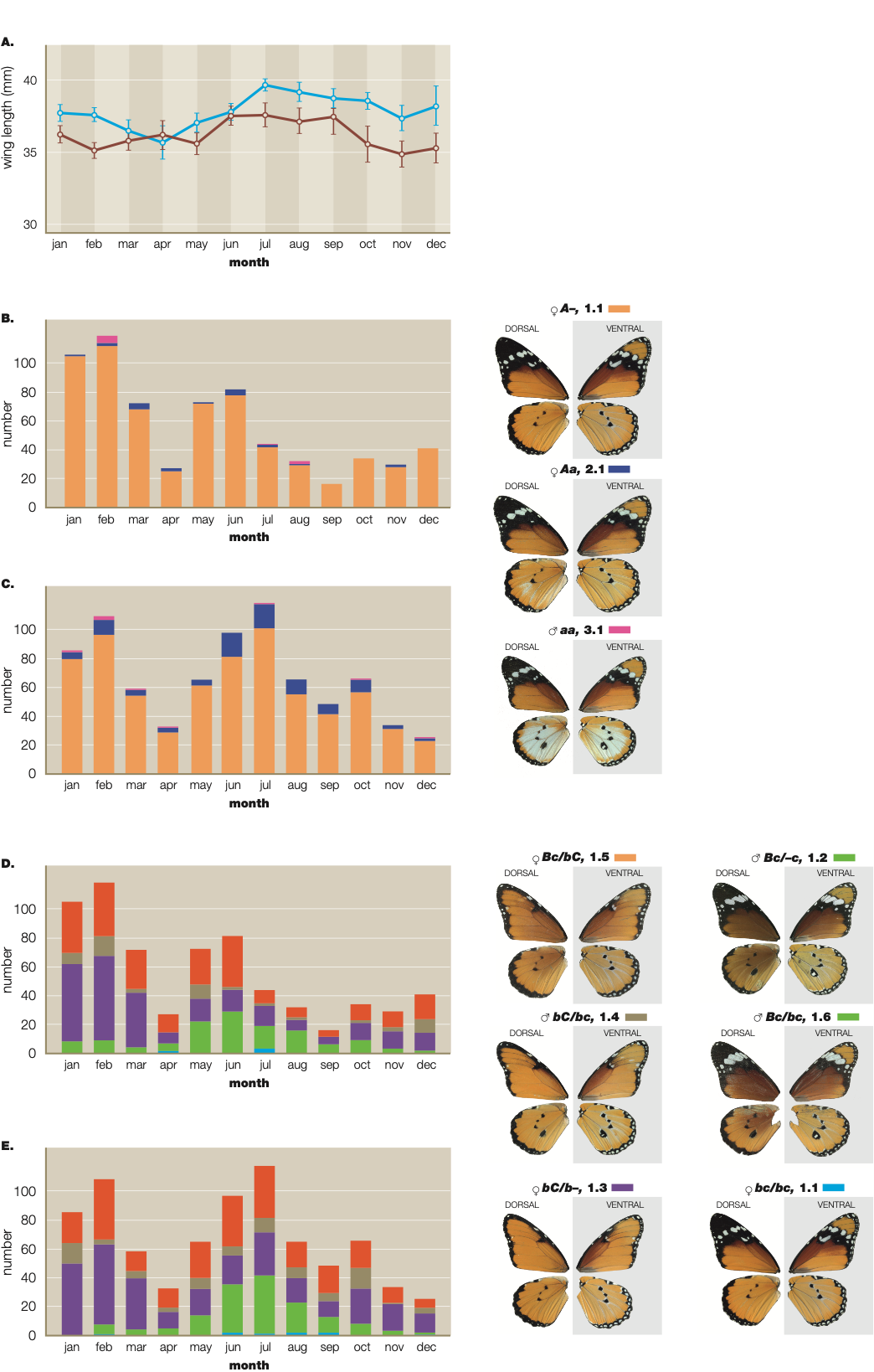


Figure S2. A. Seasonal variation in wing lengths of *D. chrysippus* – males (blue) and females (grey). Error bars show two standard errors of the mean; B. seasonal frequencies of A locus genotypes in females; C. seasonal frequencies of A locus genotypes in males; D. seasonal frequencies of BC genotypes in females; E. seasonal frequencies of BC genotypes in males. All the data relate to butterflies wild-caught, marked and released on the University campus at Dar es Salaam in 1974-75. Genotype and phenotype (Table 1) of the butterflies figured are as follows: *A-*, 1.1 ♀; *Aa*, 2.1 ♀; *aa*, 3.1. ♂; *Bc*/*bc*, 1.6 ♂; *Bc*/*bC*, 1.5, ♀; *bC*/*bc*, 1.4, ♂; *bC*/*b-*, 1.3, ♀; *Bc*/*-c*, 1.2, ♂; *bc*/*bc*, 1.1, ♀.


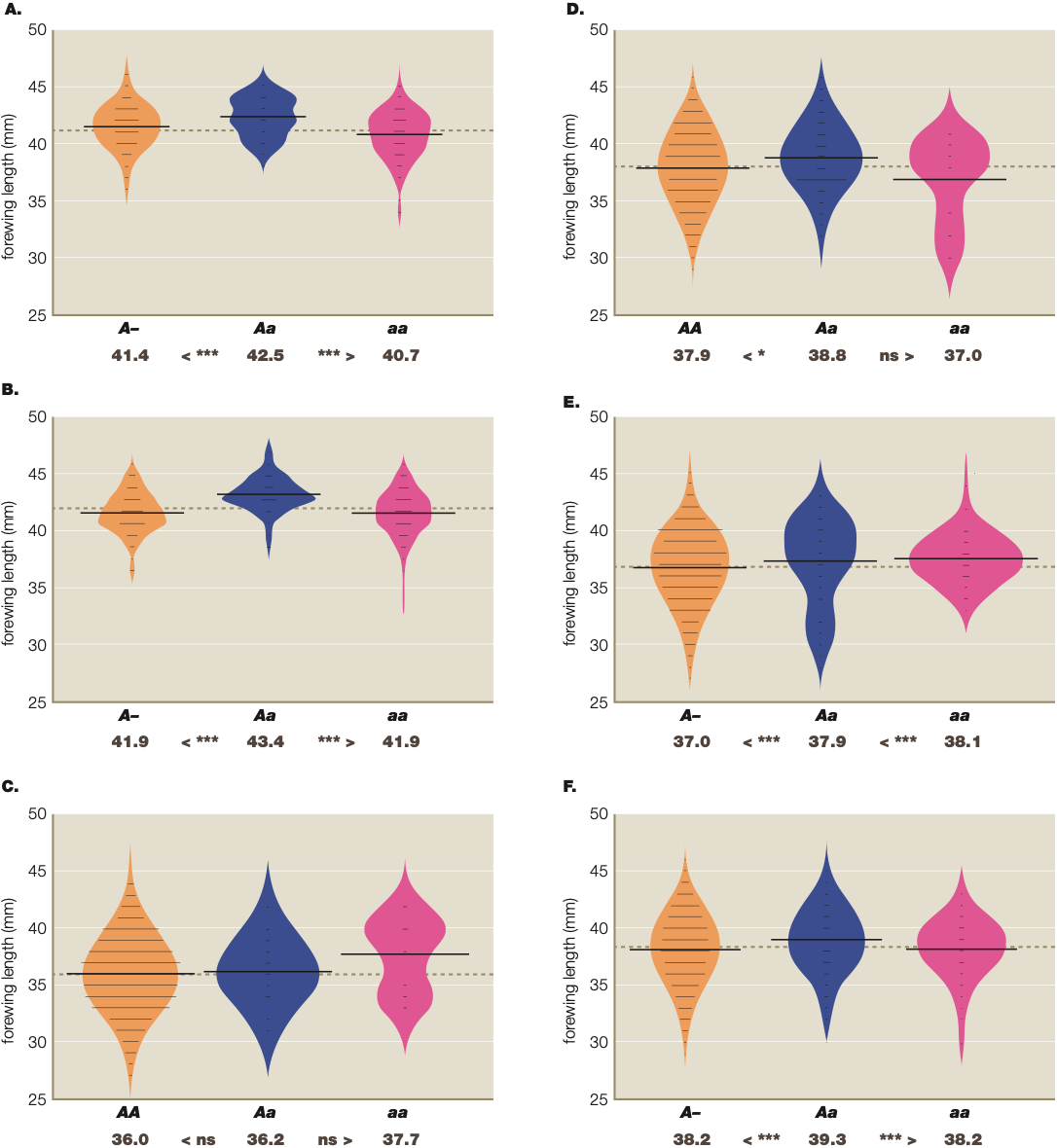
Figure S3. Beanplots showing frequency distributions of forewing lengths (mm) associated with genotypes at the A locus at Dar es Salaam. A. Lab-reared females (DB2). B. Lab-reared males (DB2). C. Wild-caught females (DB1). D. Wild-caught males (DB1). E. Wild-caught females, F wild-caught males from DB4. C and D controlled for seasonal change, E and F controlled for collecting sites but not for seasonal change.


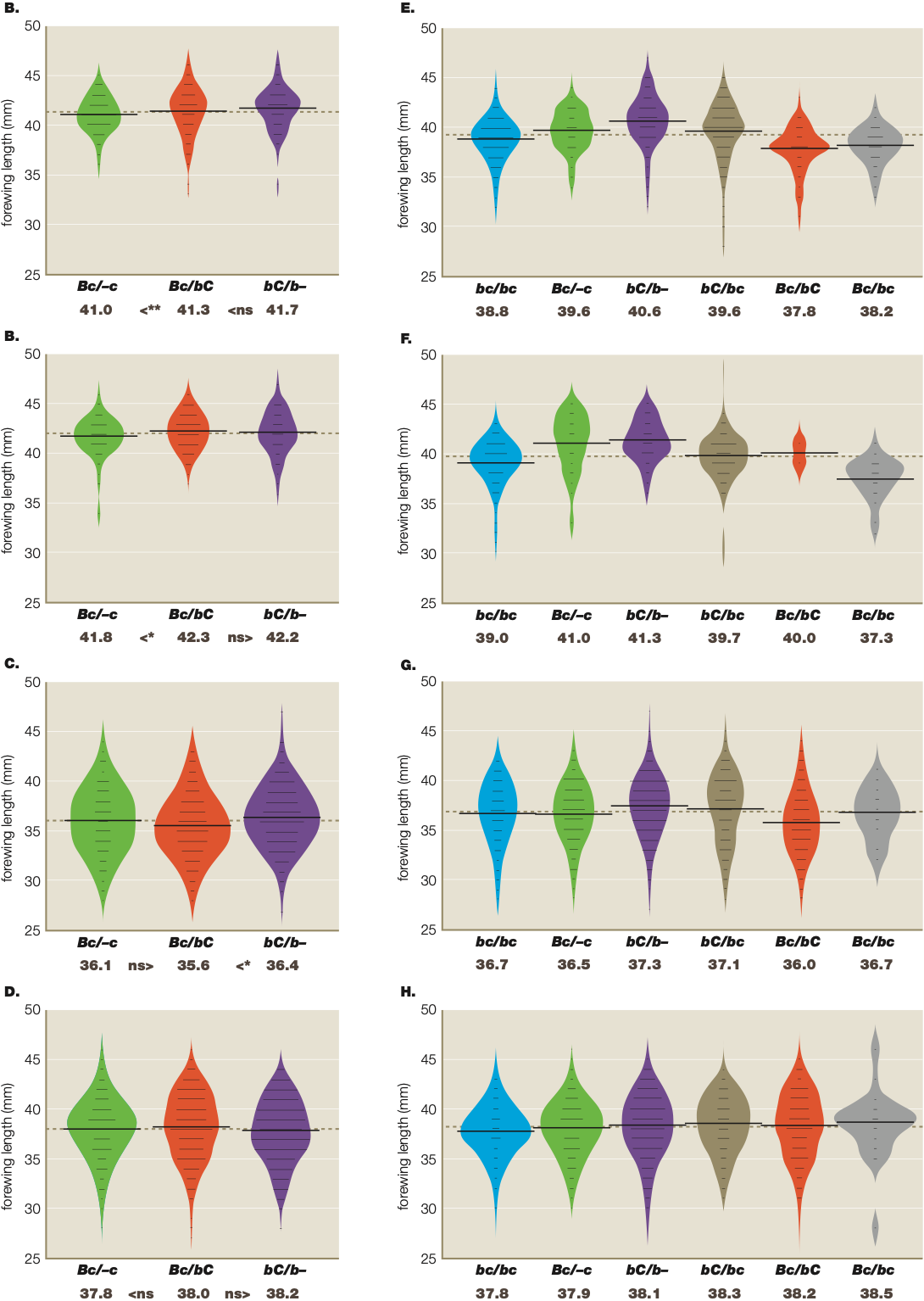


Figure S4. Beanplots showing frequency distributions of forewing lengths (mm) associated with genotypes at the BC locus.


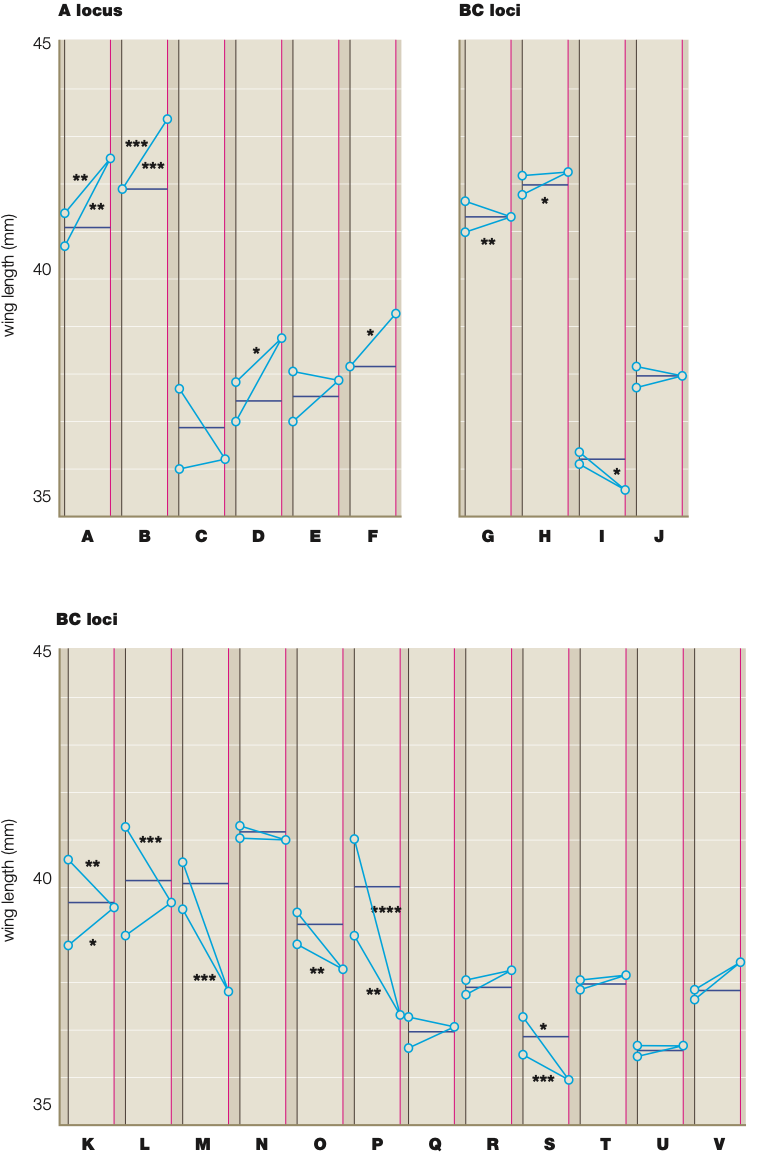
Figure S5. Graphs showing the observed wing lengths of *D. chrysippus* parents and, in laboratory crosses, F_1_ hybrids. The sexes are shown separately throughout. In wild populations the F_1_ crosses are presumptive rather than actual. Points in blue show, on the left, the mean values of the two parent genotypes and, on the right, the F_1_. Mid-parent values are marked as horizontal lines in black. Statistical significance of size differences: * *p* < 0.05, ** *p* < 0.01, *** *p* < 0.001, **** *p* < 0.0001. Differences not marked as statically significant might in some cases be so if sample sizes were larger.

(A) Female and (B) male genotypes, *A*-, *aa* (parents) and *Aa* (F_1_) in laboratory-reared crosses at Dar es Salaam, DB2., controlled for foodplant.

(C) Female and (D) male genotypes, *A*-, *aa* (parental genotypes) and *Aa* (F_1_ genotype) in a wild population at Dar es Salaam, DB1.

(E) Female and (F) male genotypes, *A*- *aa* (parental genotypes) and *Aa* (F_1_ genotype) in wild populations through the contact zone, DB3. Controlled for collecting site.

(G) Female and (H) male genotypes, *Bc*/*-c*, *bC*/*b-* (parents) and *Bc*/*bC* (F_1_) in laboratory-reared crosses at Dar es Salaam, DB2, controlled for foodplant.

(I) Female and (J) male genotypes, *Bc*/*-c*, *bC*/*b-* (parental genotypes) and *Bc*/*bC* (F_1_ genotype) in a wild population at Dar es Salaam, DB1.

(K) Female and (L) male genotypes, *bC*/*b-*, *bc*/*bc* (parental genotypes) and *bC*/*bc* (F_1_ genotype) in wild populations through the contact zone, DB4.

(M) Female and (N) male genotypes, *Bc*/*-c*, *bC*/*b-* (parental genotypes) and *Bc*/*bC* (F_1_ genotype) in wild populations through the contact zone, DB4.

(0) Female and (P) male genotypes, *Bc*/*-c*, *bc*/*bc* (parental genotypes) and *Bc*/*bc* (F_1_ genotype) in wild populations through the contact zone, DB4.

(Q) Female and (R) male genotypes, *bC*/*b-*, *bc*/*bc* (parental genotypes) and *bC*/*bc* (F_1_ genotype) in laboratory-reared butterflies reared from wild collected eggs in the contact zone, DB3 controlled for foodplant.

(S) Female and (T) male genotypes, *Bc*/*-c*, *bC*/*b-* (parental genotypes) and *Bc*/*bC* (F_1_ genotype) in laboratory-reared butterflies reared from wild collected eggs in the contact zone, DB3, controlled for foodplant.

(U) Female and (V) male genotypes, *Bc*/*-c*, *bc*/*bc* (parental genotypes) and *Bc*/*bc* (F_1_ genotypes) in laboratory-reared butterflies reared from wild collected eggs in the contact zone, DB3, controlled for foodplant.


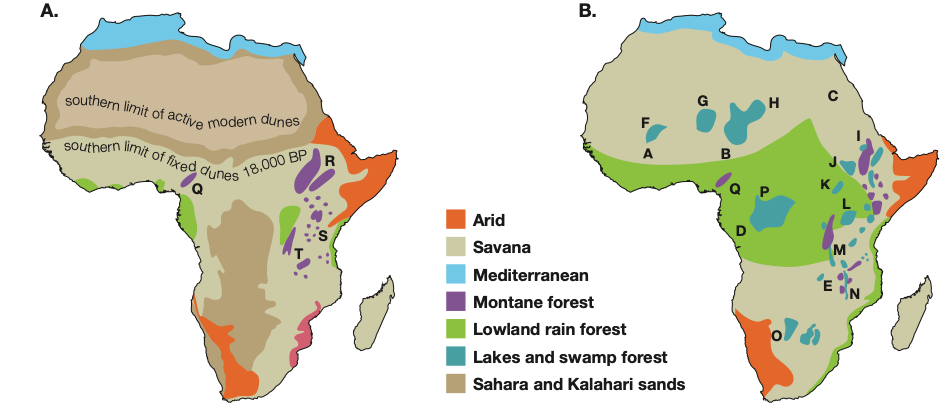


Figure S6. A. African vegetation at the maximum extent of Pleistocene glaciation, 18 ka BP. Q, Mount Camaroun; R, Ethiopian Highlands; S, Kenya Highlands; T, Tanzanian Highlands. B. African vegetation from ~15-5 ka BP in the Holocene, known as the African Humid Period (AHP). A, River Niger; B, River Benue; C, River Nile; D, River Congo; E, River Zambezi; F, Araoune Basin; G, Ténére Basin; H, Lake MegaChad; I, Lake Tana; J, Sudan Swamp; K, Lake Turkana; L, Lake Victoria; M, Lake Tanganyika; N, Lake Malawi; O, Okavango Delta; P, Lake Zaire. Vegetation maps for former glacial periods would resemble 6A. A vegetation map for the last interglacial (Eemian) Period, 130 – 115 ka BP (marine isotope stage 5e), is virtually identical to 6B, as are vegetation reconstructions for many former interglacial and interstadial periods in the Pleistocene.

**Supplementary references**

Higgins LG, Riley ND. 1970. *A Field Guide to the Butterflies of Britain and Europe*. Glasgow: Collins.

Smith, DAS. 2014. *African Queens and their Kin: A Darwinian Odyssey*. Taunton: Brambleby Books.
